# Global and local feature fusion *via* long and short-term memory mechanism for dance emotion recognition in robot

**DOI:** 10.3389/fnbot.2022.998568

**Published:** 2022-08-24

**Authors:** Yin Lyu, Yang Sun

**Affiliations:** ^1^College of Music, Huaiyin Normal University, Huai'an, China; ^2^College of Software, Shenyang Normal University, Shenyang, China

**Keywords:** dance emotion recognition, robot, LSTM, feature fusion, linear prediction coefficient

## Abstract

In recent years, there are more and more intelligent machines in people's life, such as intelligent wristbands, sweeping robots, intelligent learning machines and so on, which can simply complete a single execution task. We want robots to be as emotional as humans. In this way, human-computer interaction can be more natural, smooth and intelligent. Therefore, emotion research has become a hot topic that researchers pay close attention to. In this paper, we propose a new dance emotion recognition based on global and local feature fusion method. If the single feature of audio is extracted, the global information of dance cannot be reflected. And the dimension of data features is very high. In this paper, an improved long and short-term memory (LSTM) method is used to extract global dance information. Linear prediction coefficient is used to extract local information. Considering the complementarity of different features, a global and local feature fusion method based on discriminant multi-canonical correlation analysis is proposed in this paper. Experimental results on public data sets show that the proposed method can effectively identify dance emotion compared with other state-of-the-art emotion recognition methods.

## Introduction

Today is an era of artificial intelligence technology explosion, the demand for human-computer interaction (HCI) technology (Yu et al., 2020; Liu et al., 2022) is also increasing. Among them, emotion recognitionis an indispensable part of this technology. Facial expression is an important signal of a person's emotional state. Together with speech, hand and body posture, it forms the basic communication system of human beings in social environments. Whether we can provide perfect service for human beings according to human emotions, the key problem is to accurately identify human emotions, so as to meet human needs more intelligently (Chowdary et al., 2021; Kashef et al., 2021). Therefore, the direction of emotion recognition attracts many scholars to conduct research.

Of course, human emotions not only contain facial expressions. In real life, people can express their emotions in various forms, such as voice information, music information, physiological signals and text information, etc., which are more conducive to emotion recognition to some extent (Abbaschian et al., 2021). For example, when people is excited, people speak faster and may be accompanied by dancing gestures; When they are sad, people will droop their face and eyes, speak slowly, and may support their face with their hands. At this point, when the emotion occurs, it will also cause a certain degree of physiological changes. In addition, the corresponding emotions can also be identified through text messages. It is not enough to identify emotions only by one feature. Human emotions are inherently diversified, and features extracted by multiple modes of multiple features are more comprehensive.

Emotions can be recognized from the so-called body language, face-play, and speech. Most of their characteristics are changing with age, education, experience, etc. Moreover, there is variability among speakers, their body language, and facial expressions (Kacur et al., 2021). Emotion recognition can greatly promote the integration and development of many different disciplines, such as graphics and image processing, artificial intelligence, human-computer interaction and psychology (Jiang and Yin, 2021; Shen et al., 2021). In human-computer interaction scenes with many different modes, the combination of emotion, posture, sound and other modes can make human-computer interaction experience more real. In addition, the study of dance emotion has great application value in many fields. For example:

1. Game development. Game developers can identify players' facial expressions and determine the preferences of the majority of players, so that they can change the design scenario, difficulty or scheme of a game to provide a better experience for players.
2. Online teaching. Through the terminal operating system real-time acquisition of the students in the class facial expressions, timely detection of students interested in the teacher teach content, state of the students in class lectures are in good condition (Yu, 2021), whether the student to the teacher speak content understanding and grasp, and can identify to feedback the result to the teaching system, so convenient teacher in time according to the results of the identification of teaching activities and scheme adjustment to develop more effective learning strategies for students.
3. Safe driving. Sensors installed in the car can monitor the owner's facial expressions in real time, detect the current driving state of the driver, if the driver is detected in the state of fatigue driving, will timely alarm sound, remind the driver to stay awake, to avoid the occurrence of tragedy.
4. Medical system (Chen et al., 2021a). Design a medical machine that can recognize facial expressions, and timely tracking and detecting the patients' facial expressions. When a patient's facial expression is recognized as pain, the machine system can sound an alarm to call the medical staff, so that the patient care is more efficient, more intelligent and humane.

The emotional features contained in voice signals in audio can be expressed from the speaker's pitch, accent weight and speed, etc. Audio features reflecting certain emotions can be roughly divided into three categories: spectral features, prosodic features and tone quality features (Wang and Wang, 2021). Most of the methods to identify emotions through speech signals adopt the prosodic features of sound, among which the fundamental frequency and amplitude of sound are the most effective for emotion recognition (Murugappan and Mutawa, 2021). However, in the actual research process, it is not accurate to make judgment only by using a certain feature. The characteristics of speech emotion are not only prosodic, but also tone quality and spectrum. Asghar et al. (2022) proposed that using amplitude and frequency spectral features (MSFs) and mel-frequency cepstral coefficients (MFCCs), perceptual weighted linear predictive (PLP) and perceptual features had achieved good speech emotion recognition effects. Chouhan et al. (2021) used CNN and SVM to classify and recognize speech emotion and achieved good results. Kaur and Kumar (2021) adopted CNN for speech emotion recognition in the data set, which greatly improved the ability of speech emotion recognition. At present, the popularity of CNN model is also applied in the field of speech emotion recognition, including short and long short-term memory network (LSTM), recurrent neural network (RNN) (Yadav et al., 2021), etc,. Mohanty and Palo (2020) proposed to extract prosodic and spectral parameters of audio, and then used probabilistic neural network (PNN) and hidden markov model (HMM) to extract prosodic and spectral parameters of audio. HMM processed these two kinds of parameter features (Dai et al., 2021).

At present, there are two kinds of emotion feature extraction methods: static texture feature based and dynamic texture feature based. Emotion recognition based on static texture features is to extract the key frame of the video expression first, and replace the whole video expression recognition result with the key frame recognition result. Although it improves the speed of emotion recognition and eliminates a lot of redundant information, it lacks the time domain information. The method based on dynamic texture features contains this time domain information, and its research data is a video sequence or dynamic video. Feature extraction methods based on static texture are representative of PCA, LDA, ICA, LBP, Gist, and Gabor transform (Karim et al., 2019; Shafiq et al., 2021; Yin et al., 2021). Feature extraction methods based on dynamic texture include LBP-TOP, PHOG-TOP, LPQ-TOP, etc.

At present, although great progress has been made in the field of dance emotion recognition, there are still some problems that can not be ignored, that is, low recognition efficiency, different results with disunity of database. In order to better solve the problems faced by dance emotion recognition and further improve its practical application, this paper proposes a new dance emotion recognition based on global and local feature fusion method.

The structure of this paper is organized as follows. Section “Proposed dance emotion recognition” introduces the proposed dance emotion recognition method in detail. Then, we conduct rich experiments for the proposed method in section Experiments and analysis. There is a conclusion in section Conclusion.

## Proposed dance emotion recognition

The occurrence of dance emotion is a dynamic process, which contains both time domain information and space domain information. Considering the temporal and spatial characteristics of audio features, a new feature extraction algorithm based on linear prediction Mayer frequency cepstrum coefficient (LPMFCC) is proposed. At present, the extraction of dance emotional features is mostly based on a single voice feature, which can only reflect one attribute of voice information, not the global information of expression, and the dimension of data features is very high. In this paper, we adopt LPMFCC to extract the local feature and LSTM to extract global feature. Considering the complementarity of different features, this paper proposes a dance emotion recognition system based on global and local feature fusion. The adopted feature fusion method in this paper is the latest feature fusion framework based on kernel entropy component analysis+ discriminant multiple canonical correlation analysis (KECA + DMCCA).

KECA works by projecting raw data into higher-dimensional space to Eigen decomposing the Kernel matrix (Chen et al., 2021b). The eigenvector with the maximum eigenvalue is selected to form a new data space. It is underpinned by Renyi entropy and Parzen window. KECA can resolve the problem of the linear inseparability of the other model and enhances the separability between features.

**Figure 1:**
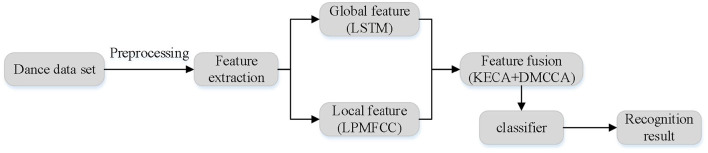
Flow chart of proposed dance emotion recognition.

The framework diagram of proposed dance emotion recognition in this paper is shown in Figure 1. The proposed algorithm includes three main steps: preprocessing, feature extraction and classification. The feature extraction process extracts a set of global features and a set of local features respectively. After feature extraction, feature dimension is higher and invalid information is more, so the effective feature fusion framework KECA+DMCCA is adopted after feature extraction. This framework can not only fuse multiple groups of information, but also greatly reduce the feature dimension.

### Dance emotion preprocessing

In order not to cause a lot of information redundancy, and not to lose the corresponding key information, but also to retain certain emotional time domain information, here, we adopt a face detection scheme based on the HSV color model (Bobbadi et al., 2022). In the HSV color model, H and S components represent color information, and V represents brightness information. The HSV color model is closely related to human's intuition on color. The RGB component of an image can be converted to HSV color space using the following formula:


(1) $$\begin{array}{l} H=\left\{H_{1}\;if\;B\leq G;360^{{}^{\circ}}-H_{1}\;if\;B>G\right\} \end{array}$$

where


(2) $$\begin{array}{l} H_{1}=\cos^{-1}\left\{\frac{0.5\left[\left(R-G\right)+\left(R-B\right)\right]}{\sqrt{{\left(R-G\right)}^{2}+\left(R-B\right)\left(G-B\right)}}\right\} \end{array}$$

(3) $$\begin{array}{l} S=\frac{\max\left(R,G,B\right)-\min\left(R,G,B\right)}{\max\left(R,G,B\right)} \end{array}$$

(4) $$\begin{array}{l} V=\frac{\max\left(R,G,B\right)}{255}. \end{array}$$

We use the plane envelope approximation (Lee and Pietruszczak, 2021) to approximate human skin color. In the planar envelope method, a pixel is considered a skin pixel if its color meets the following two conditions:


(5) $$\begin{array}{l} S\geq Th_{s};S\leq -H-0.1V+110\\ H\leq -0.4V+75;V\geq Th_{v} \end{array}$$

(6) $$\begin{array}{l} IfH\geq 0,S\leq 0.08\left(100-V\right)H+0.5V \end{array}$$

(7) $$\begin{array}{l} \text{Otherwise }S\leq 0.5H+35 \end{array}$$

### LPMFCC for local feature extraction

Linear prediction is a common method for speech analysis. It can not only get the prediction waveform of speech signal, but also provide a very good channel model. The main idea is that there is correlation between sampling points of speech signal. The sampled values of the speech signal at a certain time can be approximated by the linear combination of the sampled values at the previous time so that the waveform of the speech signal can be estimated and predicted. In order to determine the linear prediction coefficient of speech samples, it is necessary to minimize the mean square error between the linear prediction sample value and the actual speech sample value. The linear prediction coefficient reflects the characteristics of speech signal.

According to the above ideas, the linear prediction coefficient is calculated. After preprocessing the speech signal, the *p*-order linear prediction is to predict the sampling value {*s*(*n* − 1), *s*(*n* − 2), ⋯, *s*(*n* − *p*)} at this moment by using the linear combination of sampling values at the previous p times of the speech signal *s*(*n*), and the obtained prediction signal ŝ(*n*) is:


(8) $$\begin{array}{l} ŝ\left(n\right)=\overset{p}{\underset{k=1}{\sum }}a_{k}s\left(n-k\right) \end{array}$$

where *a*_*k*_ is the linear prediction error formed by the linear prediction coefficient.


(9) $$\begin{array}{l} e\left(n\right)=s\left(n\right)-ŝ\left(n\right)=s\left(n\right)-\overset{p}{\underset{k=1}{\sum }}a_{k}s\left(n-k\right). \end{array}$$

In order to optimize the prediction effect, it is necessary to minimize the mean square value of the prediction error. The formula for the mean square value of the prediction error is:


(10) $$\begin{array}{l} \varepsilon =E\left[e^{2}\left(n\right)\right] \end{array}$$

In order to minimize the mean square value of the prediction error, it is necessary to take the partial derivative of the mean square value of the prediction error formula and make it zero, as shown in Formula (11).


(11) $$\begin{array}{l} \frac{\partial \left[e^{2}\left(n\right)\right]}{\partial a_{k}}=0,k=1,2,\cdots \;,p \end{array}$$

And we can get:


(12) $$\begin{array}{l} s\left(n-i\right)\left(n\right)=\overset{p}{\underset{k=1}{\sum }}a_{k}s\left(n-k\right)s\left(n-i\right),i=1,2,\cdots \;,p \end{array}$$

If we define:


(13) $$\begin{array}{l} \varphi \left(i,k\right)=s\left(n-i\right)s \end{array}$$

Then equation (12) can be changed as the formula (14).


(14) $$\begin{array}{l} \varphi \left(i,0\right)=\overset{p}{\underset{k=1}{\sum }}a_{k}\varphi \left(i,k\right),i=1,2\cdots p \end{array}$$

**Figure 2:**
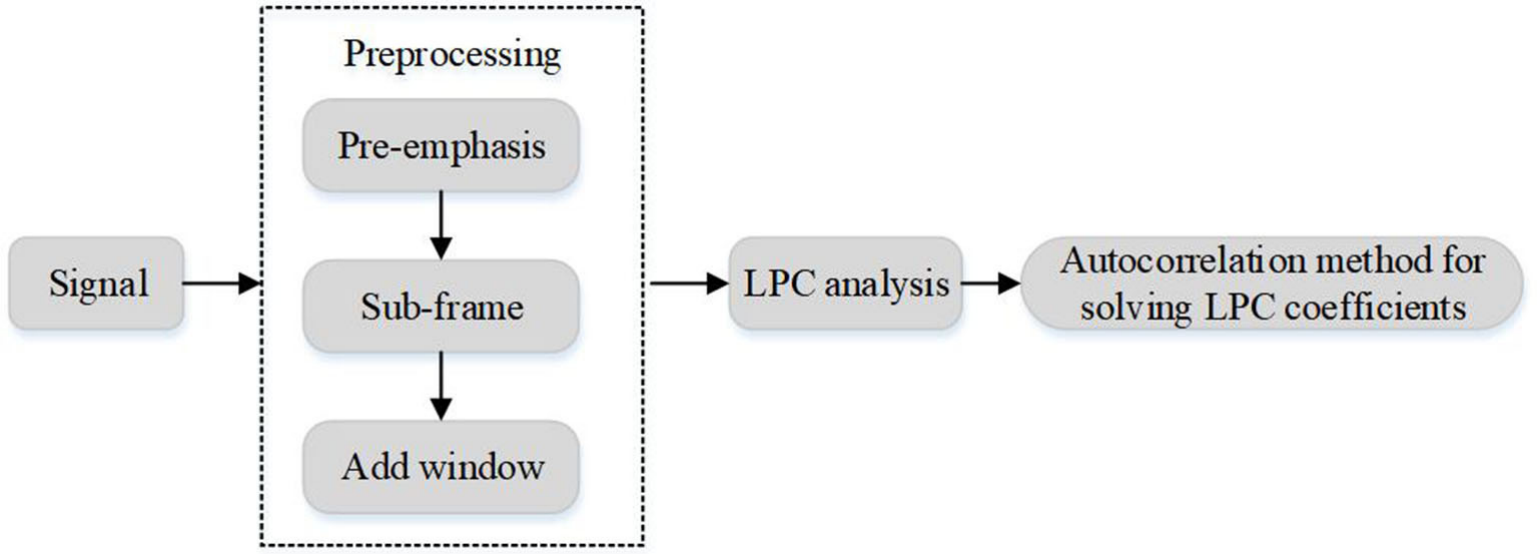
LPC coefficient extraction process.

Obviously, the linear prediction coefficient *a*_*k*_ can be obtained by solving the equation obtained by Formula (14). In this paper, the auto-correlation method and Levinson-Durbin recursion method are used to solve the equations. The prediction coefficients obtained by the above algorithms represent the feature vectors of speech frames, namely LPC feature parameters, and its extraction process is shown in Figure 2.

Linear prediction Mayer frequency cepstrum coefficient is a new characteristic parameter combining LPC and MFCC characteristic parameters. LPC parameters reflect the linear characteristics of speech, but have the disadvantage of being greatly disturbed by environmental noise. The MFCC parameters reflect the nonlinear characteristics of speech, and transform the actual frequency of speech to the Merle frequency that conforms to the auditory characteristics of human ear (Sirimontree et al., 2021). When the actual frequency is <1 kHz, the relationship between Mayer frequency and actual frequency is approximately linear. When the actual frequency is >1 kHz, the relationship between the Meir frequency and the actual frequency can be approximated as a pairwise number. The general expression of the relationship between Mayer frequency and actual frequency is:


(15) $$\begin{array}{l} f_{mel}=2958\cdot \log_{10}\left(1+f/700\right) \end{array}$$

**Figure 3:**
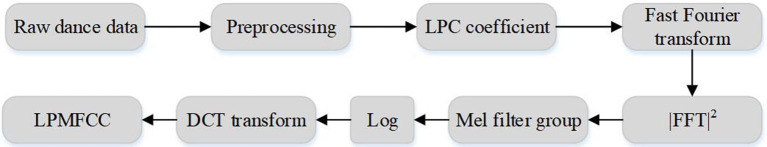
LPMFCC parameter feature extraction process.

Where *f*_*mel*_ represents the Mayer frequency and *f* represents the actual frequency. Figure 3 shows that MFCC parameters are relatively sensitive to the low-frequency part of speech. However, ambient noise is in the high frequency part of speech. Therefore, MFCC parameters have strong anti-interference ability and good robustness to environmental noise. The LPMFCC parameter is actually the LPC cepstrum parameter that converts the LPC parameter into Meyer frequency.

The LPMFCC feature extraction of speech first needs to extract the LPC coefficient of speech. After the preprocessing of speech signal *x*(*n*), such as pre-emphasis, subframe and adding window, the LPC coefficient *x*_*a*_(*n*) of each speech frame is calculated. The order of the LPC coefficient should be set equal to the number of voice samples in a frame. Secondly, the cepstrum of LPC coefficient is calculated on Meyer frequency. First, Fourier transform is made for LPC coefficient, then LPC coefficient is executed by DFT to obtain the corresponding discrete spectrum *X*_*a*_(*k*), namely:


(16) $$\begin{array}{l} X_{a}\left(k\right)=\overset{N-1}{\underset{n=0}{\sum }}x_{a}\left(n\right)e^{-\left(j2pnk/N\right)},0\leq k\leq N-1. \end{array}$$

We will take square amplitude spectrum calculation for *X*_*a*_(*k*), and obtain the discrete energy spectrum $|X_{a}\left(k\right){|}^{2}$. Where *N* is the point number of the Fourier transform. Then a set of meyerscale triangular filters are used to filter the discrete energy spectrum. The logarithmic operation is performed on the output result to obtain the logarithmic energy *Z*_*a*_(*m*), and the formula is as follows.


(17) $$\begin{array}{l} Z_{a}\left(m\right)=In\left(\overset{N-1}{\underset{k=0}{\sum }}|X_{a}\left(k\right){|}^{2}H_{m}\left(k\right)\right),0\leq m\leq M \end{array}$$

The *H*_*m*_(*k*)(0 ≤ *m* ≤ *M*) is a number of band pass filter. *M* is the number of filters. Finally, a new characteristic parameter LPMFCC is obtained by calculating the logarithmic energy by discrete cosine transform.


(18) $$\begin{array}{l} C_{a}\left(n\right)=\overset{M-1}{\underset{m=0}{\sum }}Z_{a}\left(m\right)\cos\left[\frac{pn\left(m+0.5\right)}{M}\right]. \end{array}$$

To sum up, it can be seen that the calculation method of LPMFCC characteristic parameters refers to the calculation method of MFCC coefficient and carries out cepstrum calculation of LPC coefficient under Mayer frequency. The specific extraction process is shown in Figure 3. In addition, the LPMFCC feature *Y*_*i*_ extracted from voice signal *S*_*i*_ is denoted as *Y* = {*Y*_1_, ⋯  , *Y*_*T*_}. The average eigenvector Ŷ is used to represent the features of speech signal *S*, where $Ŷ=\frac{1}{T}\overset{T}{\underset{t=1}{\sum }}Y_{t}$. T represents the frame number of speech signal S.

### LSTM for global feature extraction

In this section, by constructing the basic model of distorted FRI signals, the characteristic sequence of distorted signals is determined to be the weighted sum of multiple copies of different delay in original signals. Therefore, LSTM network is considered to be used to construct an auto-encoder to obtain the feature sequence estimation of distorted FRI. We design a novel LSTM to extract the global features.

#### Distorted FRI signal model

The FRI distortion signal is the weighted sum of several known pulses in different delay copies. Multipath effect is caused by echo in real scene. Therefore, distorted FRI signal *x*′(*t*) can be expressed as:


(19) $$\begin{array}{l} x^{\prime }\left(t\right)\;=\;x\left(t\right)+\overset{l-1}{\underset{i=0}{\sum }}a_{i}x\left(t-t_{i}\right) \end{array}$$

(20) $$\begin{array}{l} =\sum_{p\in Z}(\sum_{k=0}^{K-1}c_{k}\phi \left(t-t_{k}-pT_{\tau }\right)\\ +\sum_{i=0}^{l-1}a_{i}\left(\sum_{k=0}^{K-1}c_{k}\phi \left(t-t_{k}-t_{i}-pT_{\tau }\right)\right)+\varepsilon (t)) \end{array}$$

where *l* indicates that there are a total of *l* paths to reflect the original FRI signal. *a*_*i*_ represents the reflection coefficient of path *i*. *t*_*i*_ represents the delay of path *i*. ε(*t*) is additive White Gaussian noise.

The distorted FRI signal shown in Equation (19) is sent to the FRI sampling system, and *n*_*u*_ = *T*_τ_/*T*_*s*_ is defined. Sub-nyquist sampling samples obtained by the sampling system can be expressed as:


(21) $$\begin{array}{l} y_{n}=\overset{l-1}{\underset{i=0}{\sum }}a_{i}\left(\overset{K-1}{\underset{k=0}{\sum }}c_{k}\delta \left(t-t_{k}-t_{i}-pT_{\tau }\right)\right). \end{array}$$

#### LSTM network for FRI reconfiguration

**Figure 4:**
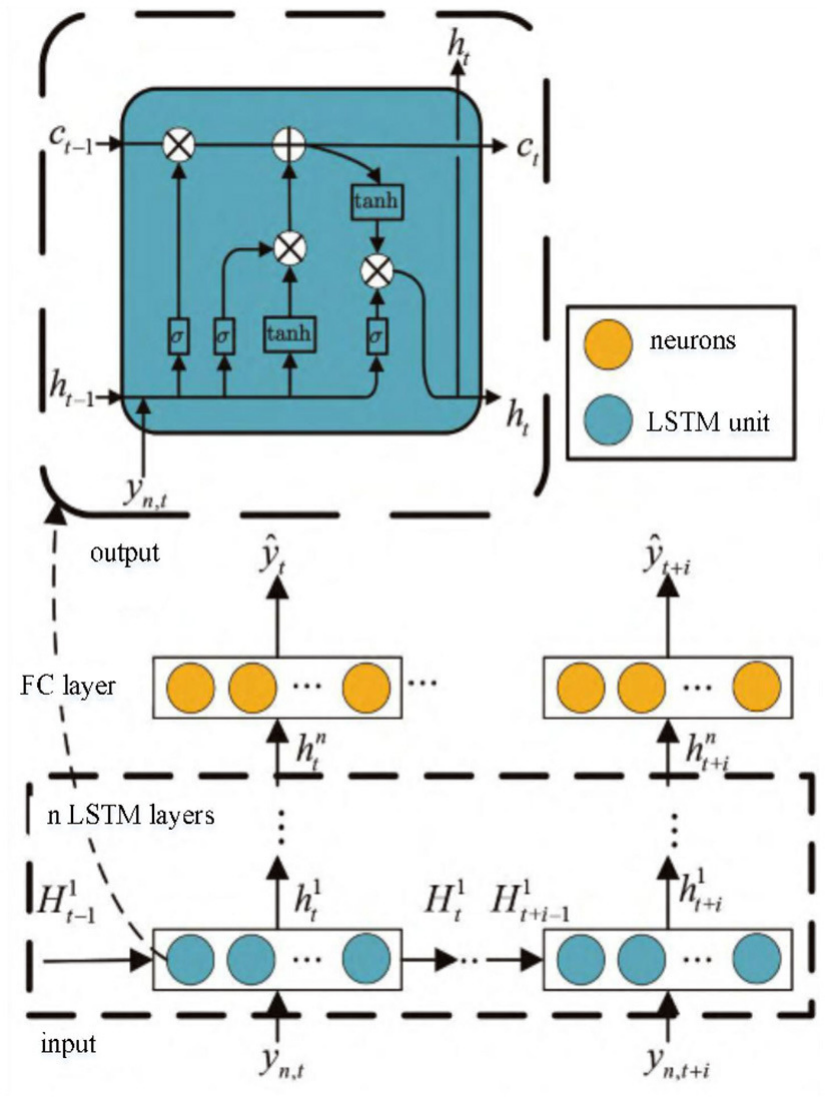
Waveform prediction model based on LSTM.

In this paper, LSTM network is considered to be used to encode FRI distorted signals, extract signal feature sequences, and train network parameters by minimizing the cost function shown in Equation (19), as shown in Figure 4. LSTM network model consists of input layer, LSTM layer, full connection layer and output layer. The input of the network is the sample *y*_*n*_ obtained from FRI sampling, and the length of the sample is *N* = 4*K* + 1. The LSTM layer is composed of several LSTM units, which mainly learn the hidden features contained in sample *y*_*n*_. The full-connection layer maps and reduces the dimension of the waveform features learned by LSTM layer, and the output layer outputs the features estimated by LSTM network. The LSTM model *f*(*W, b, y*_*n*_) is jointly determined by the three basic gate units in the LSTM structure and the cell state and output at the last moment. Under the supervision of the expected feature sequence $y_{m}^{Sinc}$, according to the cost function shown in Equation (19), parameters W and b in the model can be updated by the stochastic gradient descent algorithm to obtain the mapping weighting coefficient $\beta_{m,n}^{f}=\left\{\beta_{m,n}^{0},\beta_{m,n}^{l\neq 0}\right\}$ between the input sample *y*_*n*_ and the feature sequence $y_{m}^{Sinc}$.

The forgetting gate in the LSTM model determines the retention and discarding of waveform information in the cell state of the LSTM unit at the last moment (Wang et al., 2022), and reads the output *h*_*t*−1_ of the LSTM unit at the last moment and the input *y*_*n,t*_ of the LSTM unit at the current moment. The information is then filtered through the activation function *sigmoid(x)*. According to equation (20), the output of the forgetting gate can be expressed as:


(22) $$\begin{array}{l} f_{t}=\sigma \left(W_{f}\cdot \left[h_{t-1},y_{n,t}\right]+b_{f}\right) \end{array}$$

where σ represents the sigmoid function.

The forgetting gate outputs a number between 0 and 1, and controls the forgetting degree of the cell state *C*_*t*−1_ at the previous moment by multiplying it by the cell state *C*_*t*−1_ at the previous moment. When the forgetting gate output is equal to 1, it means that the cell state information of the last moment is completely retained. When the output is equal to 0, it means that the cell state information at the last moment is completely forgotten. If distorted waveform information exists in the sampled samples, the interaction between distortion free pulse and distortion pulse in FRI signal expression (20) can be fully utilized to eliminate the riding variable $\overset{l-1}{\underset{i=0}{\sum }}a_{i}\left(\overset{K-1}{\underset{k=0}{\sum }}c_{k}\delta \left(t-t_{k}-t_{i}-pT_{\tau }\right)\right)\cdot \phi^{\prime }\left(t/T_{s}-n-n_{l}\right)$ in the waveform through the selection of forgetting gate. So that distorted mode waveform information does not affect the cell state at the current time.

The input gate in the model determines to add new information to the cell state of the LSTM unit at the last moment. It reads the output *h*_*t*−1_ of the LSTM unit at the last moment and the input *y*_*n,t*_ of the LSTM unit at the current moment, activates it through the activation function Sigmoid and obtains the candidate vector through the activation function tanh(*x*) = (*e*^*x*^ − *e*^−*x*^)/(*e*^*x*^ + *e*^−*x*^). The input layer expression can be expressed as:


(23) $$\begin{array}{l} i_{t}=\sigma \left(W_{i}\cdot \left[h_{t-1},y_{n,t}\right]+b_{i}\right) \end{array}$$

(24) $$\begin{array}{l} {\tilde{C}}_{t}=\tanh\left(W_{c}\cdot \left[h_{t-1},y_{n,t}\right]+b_{c}\right) \end{array}$$

The input gate extracts $y_{m}^{Sinc}$, the characteristic sequence of delay information and amplitude information in the sample, and records the characteristics of delay information and amplitude information to generate candidate vector ${\tilde{C}}_{t}$. Delay information and amplitude information in cell state were updated through the interaction of candidate vector and input gate output. The LSTM unit decides to add part of $y_{m}^{Sinc}$ in the sample input at this time to the cell state through the combined action of candidate vector and input gate output. And update the cell state through the information of partial distorted waveform at the forgetting time of the forgetting gate, specifically expressed as:


(25) $$\begin{array}{l} C_{t}=f_{t}^{*}C_{t-1}+i_{t}*{\tilde{C}}_{t-1} \end{array}$$

The output gate determines the final output of the LSTM unit at that moment. It is determined by the updated cell state, the output of LSTM unit at the previous moment and the input at the current moment, and its expression is:


(26) $$\begin{array}{l} o_{t}=\sigma \left(W_{o}\cdot \left[h_{t-1},y_{n,t}\right]+b_{o}\right) \end{array}$$

(27) $$\begin{array}{l} h_{t}=o_{t}\cdot \tanh\left(C_{t}\right) \end{array}$$

H_*_ in Figure 4 includes cell state C_*_ and output h_*_. According to the model structure, the final result can be estimated as:


(28) $$\begin{array}{l} {ŷ}_{m}^{Sinc}=w_{1}h_{t}+b_{1} \end{array}$$

## Experiments and analysis

Based on the proposed mentioned algorithm in this paper, we use RML, SAVEE and self-built dance video database to make experiments. The RML database contains 720 samples, 480 short dance videos are used as training samples, and 240 short dance videos are used as test samples. SAVEE database has a total of 480 samples, among which there are 120 neutral expressions. This paper only studies the basic six types of expressions excluding neutral expressions. Then 240 short videos are used as training samples and 120 short videos are used as test samples in the experiment based on SAVEE database. In the experiment of self-built database, 240 short videos are used as training samples, among which 110 short videos are used as test samples. The proposed multi-feature extraction and fusion algorithm and support vector machine are used to achieve sentiment classification. All the experimental simulation environment in this paper is based on the experimental results of Windows 10 and MATLAB 2017a. The final experimental results and analysis are described in detail below.

**Figure 5:**
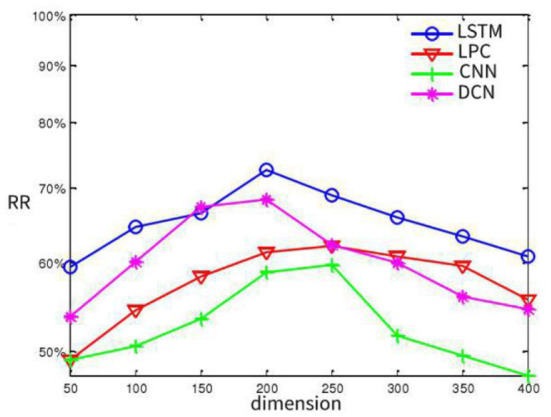
The relation between recognition rate and dimension diagram.

According to the feature extraction method mentioned above, the experiment on SAVEE database is taken as an example to determine the appropriate feature dimension reduction. We extract 1002-dimensional LSTM feature, 512-dimensional LPC feature, 753-dimensional CNN feature and 786-dimensional DCN feature. In order to fuse suitable effective features and facilitate subsequent data fusion, each feature extraction algorithm is adopted separately. Observing the relationship between dimensionality reduction and recognition rate to determine the appropriate dimensionality reduction and reduce the overall system computation, we conduct experiments on two databases respectively to observe the relationship between the highest recognition rate and the dimensionality reduction of each feature, and the results are shown in Figure 5. On the whole, it can be observed from the experimental figure that when the number of dimensionality reduction features are about 200 dimensions, the recognition rate of each feature extraction is the best.

**Figure 6:**
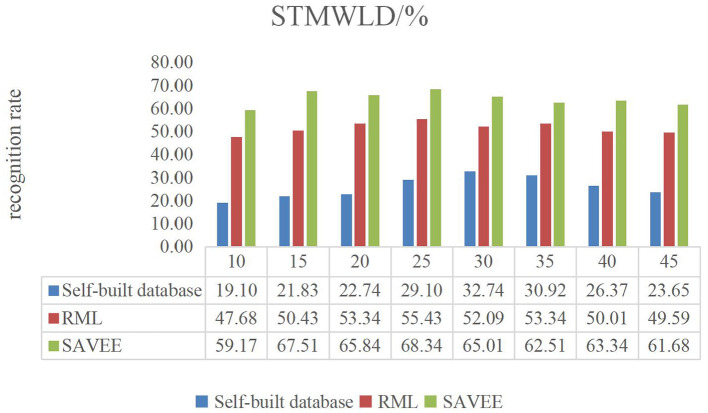
STMWLD dance video experiment result.

**Figure 7:**
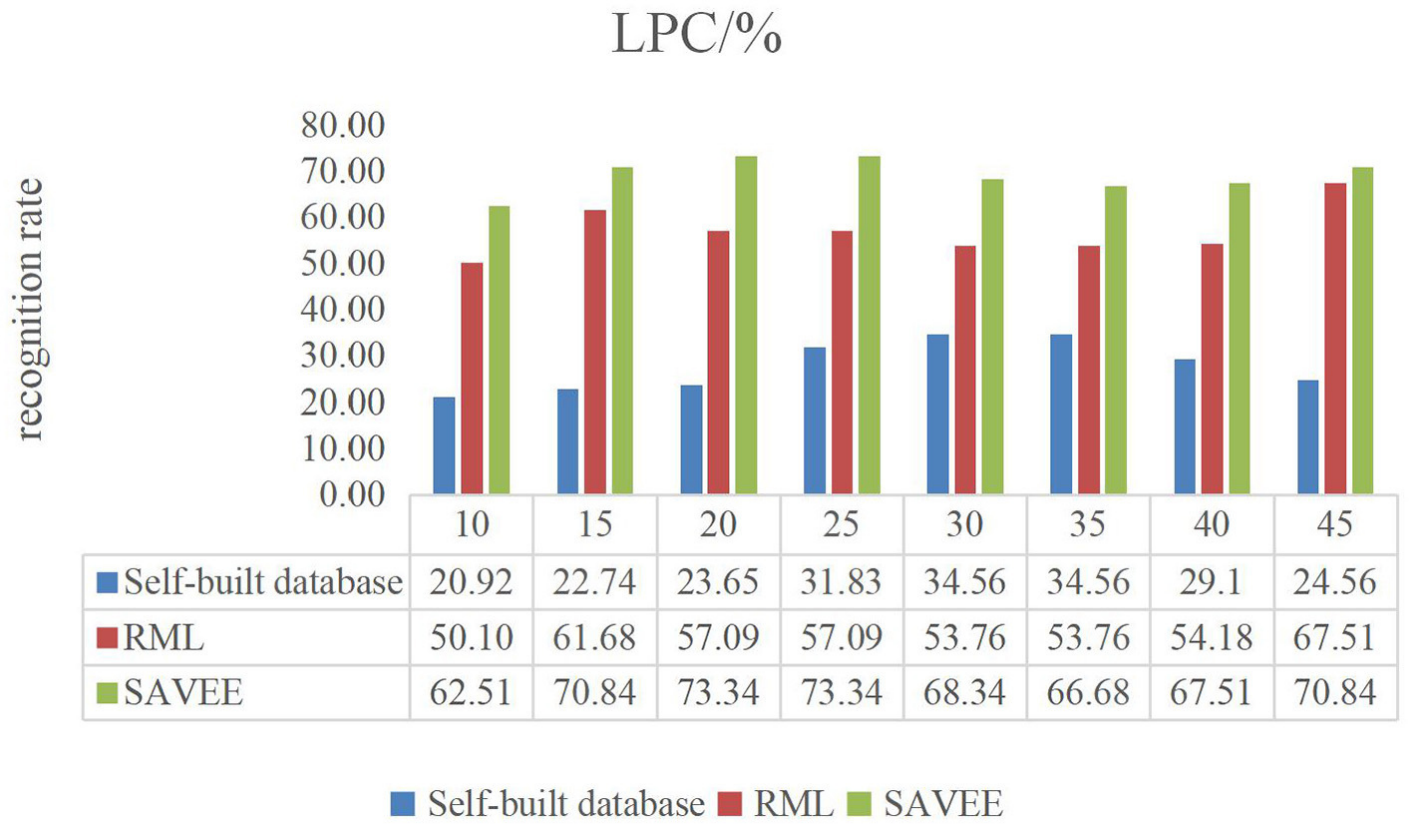
LPC dance video experiment result.

**Figure 8:**
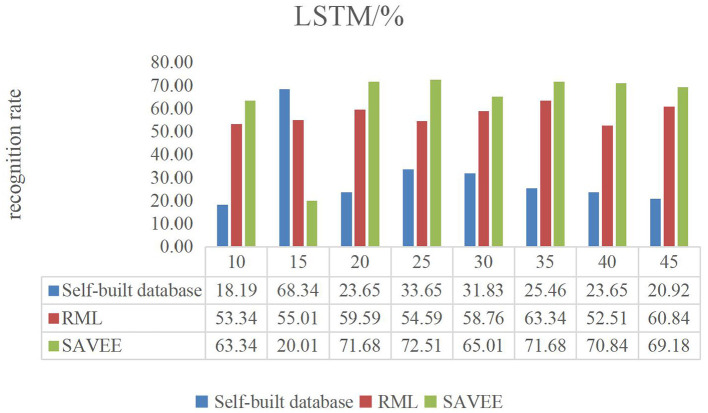
LSTM dance video experiment result.

**Figure 9:**
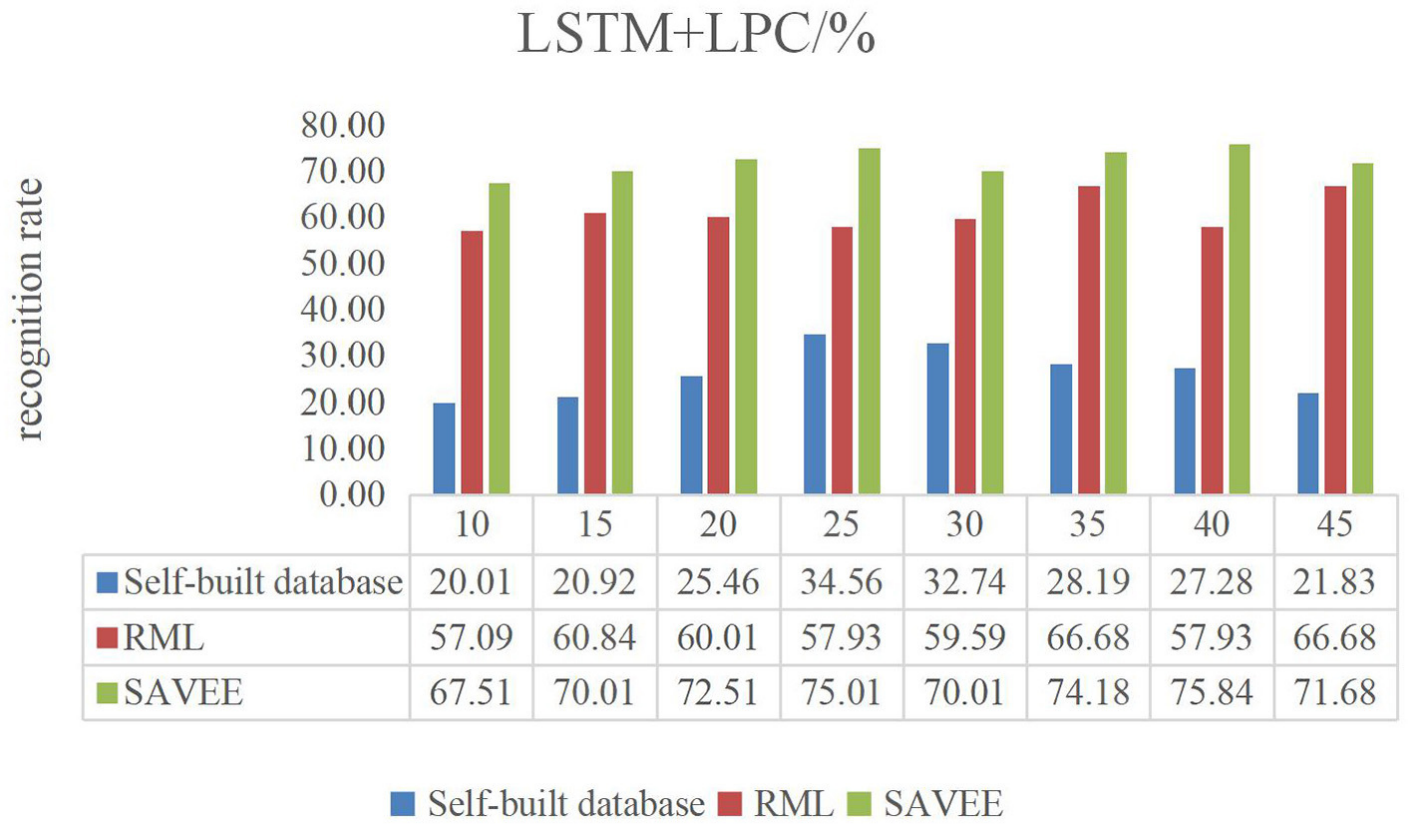
LSTM + LPC dance video experiment result.

Figures 6–**9** show the experimental results of STMWLD feature extraction algorithm alone, LPC-based local information, LSTM-based global information and LSTM + LPC based global information respectively.

The experimental results of the above four figures show the relationship between the recognition rate of dance expression and the number of beats when the dimensionality reduction is 200. In the video, one frame is selected every five frames. The purpose of selecting the frame number is to determine the maximum period of expression from the recognition rate on the one hand. On the other hand, the trend of the experimental results indicates that the occurrence of expression is a process from beginning to maximum and then to end to some extent. In general, although the feature algorithm STMWLD alone has the highest recognition rate of 32.74% in the self-built database, it has the highest recognition rate of 55.43 and 68.34% in the other two standard databases respectively, indicating the effectiveness of our proposed algorithm. The recognition rates of LPC and LSTM fusion are 34.56, 62.51, and 73.34%, respectively. When LSTM was used to extract features, the recognition results of these three databases were 33.65, 63.34, and 72.51%, respectively. The recognition rates of LSTM and LPC fusion extraction are 34.56, 66.68, and 75.84%, respectively. This also shows that different databases have a certain influence on the experimental recognition results, and the fusion of two features is better than the recognition effect of a single feature. According to the average recognition rate obtained in Figures 8, 9, the recognition rate of facial expressions using only global features is higher than that using only local features, and about 3% higher than that using only local features.

Our method is compared with other methods, including STMWLD, LPC, LPC+STMWLD, and LSTM + LPC, as shown in Table 1. As can be seen from the data in Table 1, when a single feature is adopted, some discriminative facial expression information may be lost, resulting in low recognition rate and unsatisfactory recognition effect. Compared with other methods in the table, the method proposed in this paper has the best recognition rate, which is 76.26 and 85.84% for the two databases respectively. The recognition rate of natural expressions is 55.46%, which shows the effectiveness of the proposed method in real natural scenes. The biggest advantage of this method is that it combines local features with global features, and includes dynamic time domain feature information. These complementary features are more conducive to facial expression recognition in video. On the other hand, the experimental results also demonstrate the effectiveness of the proposed method.

**Table 1:** Expression recognition rate table of each feature extraction algorithm/%.

**Feature extraction method**	**Self-built**	**RML**	**SAVEE**
STMWLD	32.74	55.43	68.34
LPC	33.65	63.34	72.51
LPC + STMWLDCNN	34.56	62.51	73.34
LSTM + LPC	**55.46**	**76.26**	**85.84**

The bold values indicate the best values obtained by proposed method.

Table 2 shows the comparison of the experimental results of feature extraction methods in this paper and those in other references. The results of experiments with different databases are also different. The table is to compare with the experimental results of feature extraction methods in other references under the condition that the selected data sets are consistent with the public data sets used in this paper as far as possible, and the relatively new references are selected for comparison with the features in this paper under the condition that the comparison standards are consistent as far as possible. It is obvious from the table that the proposed feature fusion method is superior to other feature extraction methods.

**Table 2:** Comparison between different methods and feature fusion methods in this paper.

**Method**	**Recognition rate/%**	**Recognition time/s**
HOG	35.81	4.6
CNN	63.21	2.5
Att-Net (Kwon, 2021)	75.13	2.1
CTNet (Lian et al., 2021)	76.89	1.7
CCML (Zehra et al., 2021)	73.54	1.3
Proposed	**85.84**	**0.5**

The bold values indicate the best values obtained by proposed method.

## Conclusion

Dance emotion recognition based on video is a challenging and long-term problem. The emotion in video is easily disturbed by various factors. This paper proposes an effective multi-feature fusion framework to solve the problem of video expression recognition, and studies the recognition effect of video expression in natural and real scenes. The system framework of LPC algorithm and LSTM fusing complementary and multi-feature is introduced, and then these features are fusing with KECA+DMCCA framework. Finally, SVM classifier is used to realize the recognition of six basic expressions. Experiments on two public databases (RML, SAVEE) and self-built databases prove the effectiveness of the proposed feature extraction algorithm, and the experimental results also show that the recognition effect of multi-feature fusion is better than that of single feature. In the future works, we will research more advanced deep learning methods to improve the emotion recognition.

## Data availability statement

The original contributions presented in the study are included in the article/supplementary material, further inquiries can be directed to the corresponding author.

## Author contributions

All authors listed have made a substantial, direct, and intellectual contribution to the work and approved it for publication.

## Conflict of interest

The authors declare that the research was conducted in the absence of any commercial or financial relationships that could be construed as a potential conflict of interest.

## Publisher's note

All claims expressed in this article are solely those of the authors and do not necessarily represent those of their affiliated organizations, or those of the publisher, the editors and the reviewers. Any product that may be evaluated in this article, or claim that may be made by its manufacturer, is not guaranteed or endorsed by the publisher.
